# The Sphere of Institutional Treatment

**Published:** 1913-01-18

**Authors:** 


### THE SPHERE OF INSTITUTIONAL TREATMENT.

### The "Wretched Life" of Pit Doctors.

BY A MEDICAL SUPERINTENDENT.

Some of our readers will have been amused to see, in a leading article in *The Times* of the 1st inst., the explanation that the reason why colliery medical practitioners at once accepted the Insurance Act was that they had been " better treated and more contented than those in other circumstances." Now, we take the risk of disagreeing with a journal that is nearly always admirably informed, and is, therefore, worth expert correction, and of suggesting that the real state of matters is just the opposite. As a preliminary it would be well—although the precedent might be an embarrassing one to certain schools of journalistic practice—to give our authorities. These comprise a consultant in a large North-Country city and three actual colliery practitioners. The first remarked that pit doctors had a wretched life of it, and that they all drank. To the latter part of this statement we should be very sorry to subscribe; translated from conversational hyperbole to strict fact, it means that the small percentage of medical men who take more alcohol than is good for them is noticeably higher on the coal-fields than elsewhere. The second of our informants will be recalled by those who see medical journals regularly. From the happily reached haven of a country practice in the South of England he has more than once written to the effect that his former life was well represented by the actual remark to him of one of his former patients. The remark was, that he (the doctor) was nothing but the colliers' servant, with an adjective explaining his function tacked on to his name. We made the third mental note in the course of a conversation, the expansiveness of which was helped by whiskey and soda, the whiskey, however, being in the one glass and the soda in the other. After learning that our interlocutor had been trying for some time to sell his practice, the drawback of colliery work was thus described: "The patients have no respect for one." The fourth source of information we found in an able young doctor, who had recoiled in disgust and some ill-health from one of those congeries of mean streets dotted with picture-palaces that unfortunately constitute the abode of a fair proportion of the inhabitants of Great Britain. The profits, he said, were large; a man could clear five hundred or six hundred pounds a year very soon if, as was seldom the case, he were moderately careful. The cash inducement was necessitated by the nature of the work—killing and repellent; and made practicable by the large number on the books, which, in turn, economised attention surprisingly. Serious cases one packed off to hospital, acquiring somehow a queer knack of picking them out fairly successfully at a glance. The great thing was to give medicine. A £50 drug order—stock mixtures were used of necessity—would last about three weeks. For a woman who came to him with anæmia he looked out some rather superior ferruginous capsules; she refused them indignantly and demanded a bottle of "proper medicine." Vindictively he gave her two drops of burnt sugar in eight ounces of water; and she returned the next week full of praises of its effects. His colleagues were, some of them, risking their constitutions for a few years to obtain capital with which to embark upon more congenial work; others had become like the dyer's hand; not a few, like himself, declined the work, after experience of it, altogether. With the admission that youth is not apt to under-colour its descriptions, we venture to commend all this to the attention of our respected and senior contemporary's leader-writer. What change, indeed, from existing arrangements in which anything like the above state of affairs is possible, would not be welcomed by unhappy victims? (*To be continued.*)

